# Ultrasound-based radiomics for the evaluation of breast cancer

**DOI:** 10.3389/fonc.2025.1710405

**Published:** 2026-01-12

**Authors:** Fei-Yi Sun, De-Li Meng, Lin Liu, Xiu-Qun Cao, Lu Fu, Lei Meng, Xin-Wu Cui, Xiao-Fang Pan

**Affiliations:** 1 Department of Medical Ultrasound, Health Medical Department, Central Hospital of Dalian University of Technology, Liaoning, China; 2 Department of Medical Ultrasound, Tongji Hospital, Tongji Medical College, Huazhong University of Science and Technology, Wuhan, China

**Keywords:** artificial intelligence, breast cancer, evaluation, radiomics, ultrasound

## Abstract

Breast cancer is the most common cancer among women all over the world. Ultrasound examination is instrumental in breast lesion screening, diagnosis and prognosis assessment, relying on non-radiation, inexpensiveness and real-time operation. However, it still has some limitations in diagnostic sensitivity and specificity. Radiomics aims at extracting high-throughput quantitative features from medical images, so as to deeply mine image information and further discover tumor features that cannot be discerned by naked eyes. Ultrasound radiomics models are gradually applied in evaluating breast cancer diagnosis and therapy, aiming to help with the precise diagnosis, prediction and treatment. This review summarizes the recent research progress of ultrasound radiomics in diagnosing benign and malignant breast lesion, predicting molecular subtype, lymph node status, neoadjuvant chemotherapy response and disease prognosis. Besides, the review also discusses the challenges and future research perspectives regarding ultrasound-based radiomics for the evaluation of breast cancer.

## Introduction

Breast cancer is a representative malignancy of females and also the primary cause of cancer-related deaths for females worldwide (1). Early screening, accurate diagnosis and timely treatment contributes to an improvement of patients’ overall survival rate and life quality. Ultrasound, one of the most useful imaging technologies, is widely applied in the process throughout disease diagnosis, prediction and treatment, involving diagnosing the benign and malignant of lesions, identifying lymph node metastasis (LNM) and monitoring neoadjuvant chemotherapy (NAC) responses (2–4). Technology development brings out many new ultrasound imaging techniques specific to breast screening, including ultrasound elastography and contrast-enhanced ultrasound (CEUS), which make it able to visualize different aspects of the tumors while improving the accuracy of diagnosis and prognosis prediction (5). Nevertheless, radiologists are incapable of objectively and comprehensively analyze the information obtained based on these techniques.

Radiomics assists in deeply mining medical image information by extracting relevant high-throughout quantitative features, so as to further discover tumor features unrecognizable to naked eyes (6). Recently, radiomics based on a variety of medical images (for instance, X-rays, CT, and ultrasound, etc.) has been extensively studied in terms of breast cancer (7). Ultrasound is characterized by no-radiation, inexpensiveness and real-time operation, which gives ultrasound radiomics a distinctive suited in clinical practice.

**Figure 1:**
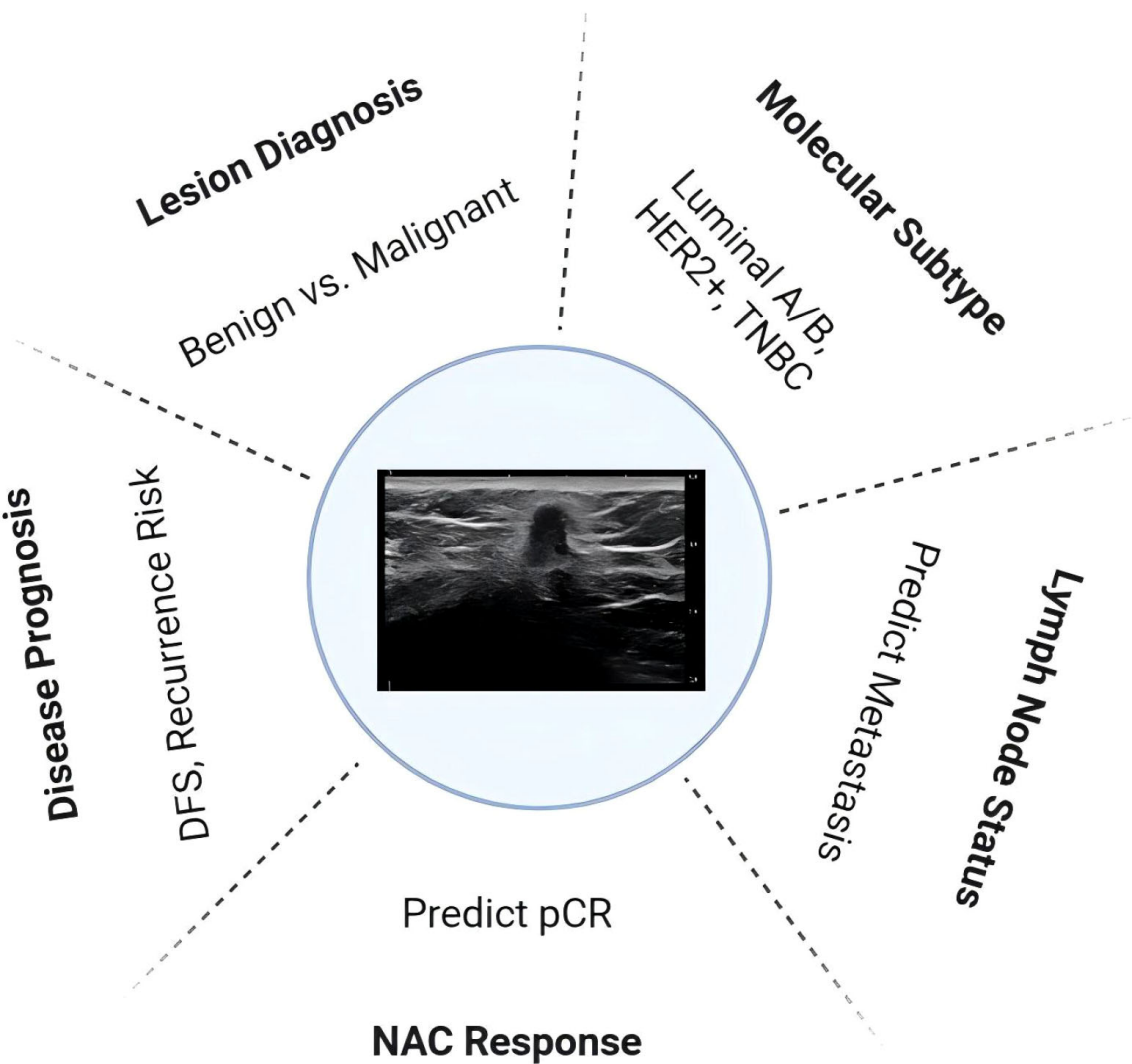
An overview of clinical application scenarios for ultrasound-based radiomics in breast cancer HER2, human epidermal growth factor receptor 2; TNBC, Triple-negative breast cancer; NAC, neoadjuvant chemotherapy; DFS, disease-free survival.

In this review, taking the clinical problems facing the diagnosis and treatment of breast cancer as the starting point, we summarize recent advances of ultrasound radiomics in diagnosing benign and malignant breast lesion, predicting molecular subtypes, evaluating lymph node (LN) status, predicting NAC responses and disease prognosis (**Figure 1**). The challenges facing radiomics together with its future research perspectives are discussed.

## Overview of radiomics

**Figure 2:**
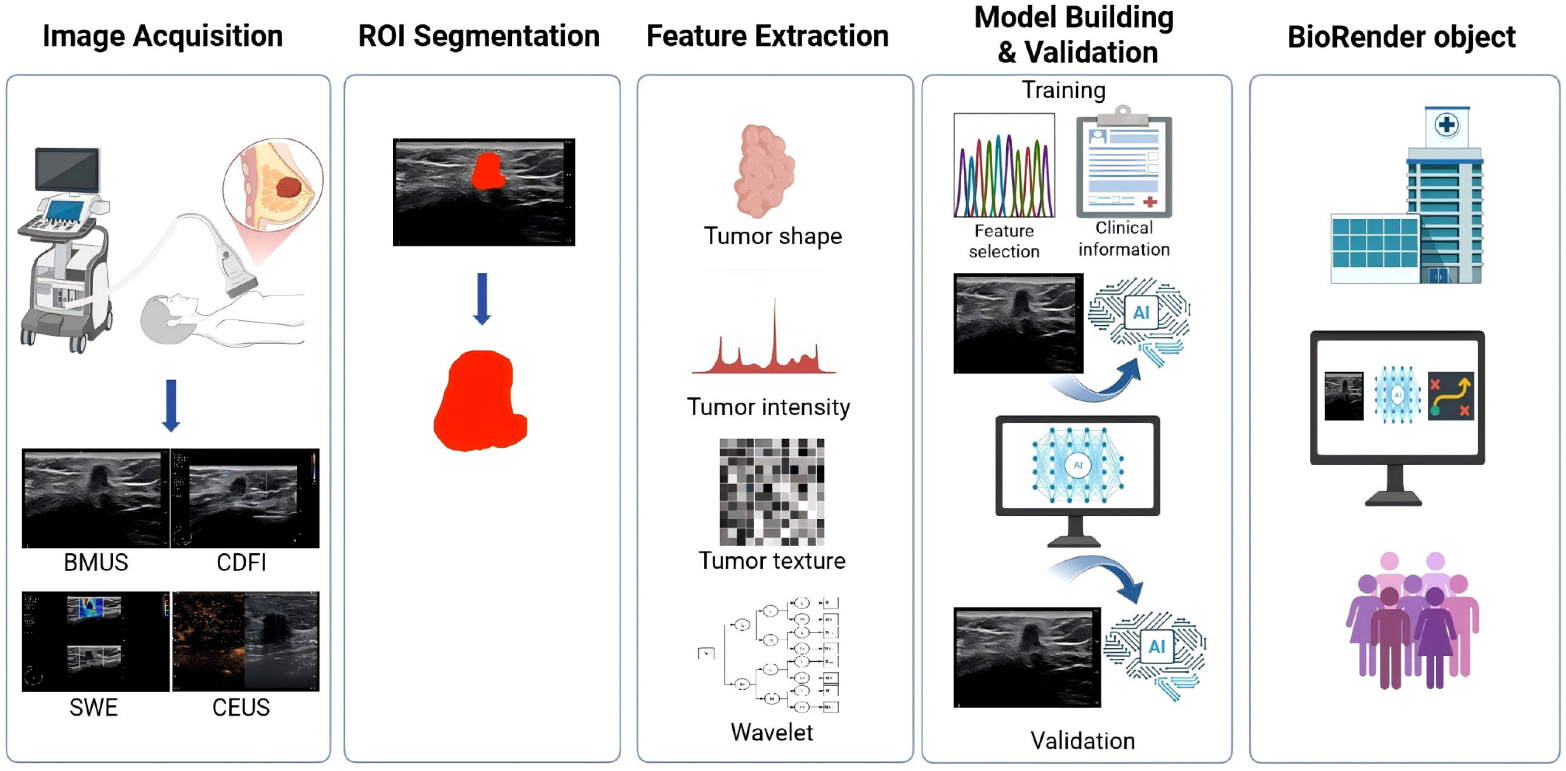
The typical workflow of ultrasound-based radiomics. BMUS, B-mode ultrasound; CDFI, color doppler flow imaging; SWE, shear wave elastography; CEUS, contrast-enhanced ultrasound; ROI, region of interest.

Radiomics, proposed by the Dutch scholar Lamnin in 2012 (6), focuses on extracting high-throughput quantitative features from medical images to assist physicians in making accurate judgments through deeper mining, predictions and analyses of large amounts of imaging data information (**Figure 2**). Relevant assumption is that the genetic- and molecular-level heterogeneity at the microscopic tissues can be expressed by their phenotypes, and that the latter can be manifested by medical images, which can then be identified through radiomics.

There are five basic steps of radiomics: 1) Medical imaging acquisition, acquiring standardized high-quality medical images is the basis for constructing reliable and stable radiomics models, the most commonly used medical images in radiomics include X-ray, CT, MRI and ultrasound. 2) Region of interest (ROI) segmentation, which is the extraction of relevant information about the lesion. In previous studies, manual segmentation of ROIs was most common and was performed by at least two experienced radiologists or experts. Researchers usually calculate the intraclass correlation coefficient (ICC) for assessing the segmentation reproducibility. In recent years, deep learning (DL) is able to recognize targets from medical images for automatic or semi-automatic segmentation, aiming to achieve higher accuracy. 3) Feature extraction, computer algorithms assist in extracting radiomics features from the drafted ROI, including morphological, texture, first-order and wavelet features. 4) Feature selection, the most representative features are selected for enhancing the model stability and accuracy by virtue of feature selection methods. Common feature selection methods are LASSO, max-relevance and min-redundancy, etc. 5) Model construction and validation, the optimal algorithm is selected for model building, then the model validation is performed using an independent external dataset.

## Ultrasound radiomics for breast lesion diagnosis

Ultrasound is a common imaging technique targeting breast cancer detection and diagnosis (5). However, a visual assessment of ultrasound will inevitably lead to the problem of inter- and intra-observer variability as well as decision-making subjectivity. Recent researches have paid attention to the exploration of the possible function of ultrasound radiomics in distinguishing benign and malignant breast lesions (**Table 1**).

**Table 1:** Summary of ultrasound radiomics in the breast lesion diagnosis.

Year	Authors	No. of lesions	Task	Modality	Result
2021	Remeo et al. (8)	201	benign vs malignant	BMUS	AUC: 0.820
2021	Shia et al. (9)	543	benign vs malignant	BMUS	AUC: 0.938
2020	Mango et al. (10)	900	benign vs malignant	BMUS	AUC: 0.870
2023	Guo et al. (11)	591	benign vs malignant	BMUS	AUC: 0.911
2023	Zhong et al. (12)	379	benign vs malignant	BMUS	AUC: 0.840
2022	Misra et al. (14)	85	benign vs malignant	BMUS+SE	Accuracy: 90%
2020	Zhang et al. (15)	291	benign vs malignant	BMUS+SWE	AUC: 1.0
2020	Youk et al. (16)	328	benign vs malignant	BMUS+SWE	AUC: 0.992 in SWE AUC: 0.929 in BMUS
2020	Moustafa et al. (17)	159	benign vs malignant	BMUS+Color Doppler	AUC: 0.958
2021	Yu et al. (18)	3623	Four categories: inflammatory masses, adenosis, benign tumors, and malignant tumors	BMUS+Color Doppler	Accuracy: 89.2%
2022	Varghese et al. (19)	131	benign vs malignant	CEUS	AUC: 0.720
2022	Zhu et al. (20)	190	benign vs malignant	CEUS	AUC: 0.750
2020	Li et al. (21)	181	benign vs malignant	BMUS+SWE+CEUS	AUC: 0.919
2022	Hong et al. (24)	496	benign vs malignant in BI-RADS US category 4 or 5	BMUS	AUC: 0.937
2022	Tang et al. (25)	577	benign vs malignant in BI-RADS US category 4	BMUS+SWE	AUC: 0.908

BMUS, B-mode ultrasound; SE, strain elastography; SWE, shear wave elastography; CEUS, contrast-enhanced ultrasound; AUC, area under the curve.

Earlier, many studies confirmed the good effect of B-mode ultrasound (BMUS) images-integrated radiomics in breast lesion diagnosis, with an AUC range of approximately 0.82-0.938 (8–10). Remeo et al. adopted a radiomics model for identifying benign and malignant nodules based on machine learning (ML) (8), which showed a higher accuracy than radiologists (82% vs 79.4%), whose performance was also improved but not significantly (p=0.508). In addition, studies showed that the model, by adding radiomics features beyond lesions, could enhance the diagnostic efficacy (11). In the study by Zhong et al., ultrasound-based intratumoral radiomic features could be employed for discriminating benign and malignant nodules (AUC:0.780), and the performance was improved by adding the peritumoral features (AUC:0.840) (12). Then, Guo et al. developed a model with different regions (intratumoral, peritumoral and ipsilateral breast parenchyma), achieved an AUC of 0.911 (11). Thus, we can confirm the presence of many valuable information in the parenchymal and peritumoral regions of breast lesions.

Ultrasound elastography, divided into strain elastography (SE) and shear-wave elastography (SWE), is a valuable supplemented tool for B-mode in breast screening, which can reflect breast lesions stiffness (13). Radiomics based on elastography has attracted attention from researchers. Misra et al. captured image features from 2D and SE to train an ensemble model built from an AlexNet model and a ResNet model (14), whose accuracy was 90% in the test set. Zhang et al. adopted a deep-learning radiomics model for extracting features from B-mode and SWE images for classifying breast lesions, reaching superior diagnostic performance than that of the BI-RADS assessment (15). Other study also yielded similar result that ultrasound radiomics based on elastography could well classify malignant and benign breast tumors (16).

For breast lesions, the blood supply characteristics are strongly correlated with malignancy. Moustafa et al. (17) and Yu et al. (18) established a radiomics model integrating color doppler flow imaging (CDFI) and gray-scale ultrasound, remarkably contributing to the breast lesion diagnosis. CEUS can offer more detailed information about the blood supply for breast lesions. Varghese et al. retrospectively collected the videos of CEUS exams from 131 women who had suspicious breast masses, with representative images obtained at 4 phases: precontrast, early, peak and delay enhancement, then the radiomics metrics were extracted from these images (19). Zhu et al. evaluated a radiomics model based on CEUS videos with a length of more than 1min, who extracted temporal and spatial information from dynamic CEUS (20). Therefore, radiomics based on CEUS static images and videos can be used to improve the diagnosis accuracy. Li et al. proposed a radiomics approach implemented attribute bagging for feature selection (21). The dataset contained the multimodal ultrasound images (BMUS, SWE and CEUS) of 178 patients. On test set, the AUC reached 0.919, the accuracy reached 84.12%, the sensitivity reached 92.86% and the specificity reached 78.80%, proving the better performance of the model than all the other methods.

The Breast Imaging Reporting and Data System (BI-RADS) serves for standardizing the terminology regarding breast lesion classification (22), which can be classified into 7 categories. Category 4 refers to suspicious lesion that has a malignant probability of 3% to 94%, and a biopsy is recommended (23). The malignancy probability of category 5 is more than 95% (22). However, some biopsies are not needed for BI-RADS 4 or 5 lesions because of the larger malignant probability. Therefore, it is in urgent need to build an accurate diagnostic model and reduce unnecessary biopsies. Hong et al. constructed a model with radiomics score and clinical predictors (BI-RADS category, maximum lesion diameter, patient age), and the AUC was 0.937 (95% CI, 0.893–0.965) for the validation cohort (24). The model also displayed excellent clinical application value, which could be used to help radiologists to differentially diagnose whether the breast lesions were classified as BI-RADS category 4 or 5. In a retrospective study, Tang et al. examined the SWE and gray-scale ultrasound images of 122 patients, confirming that the model with the maximum elasticity value reduced overdiagnosis and unnecessary biopsy in BI-RADS 4 lesion screening (25).

Different ultrasound modalities offer complementary advantages and limitations for radiomics analysis. BMUS is cost-effective, widely available, and serves as the foundational modality, yet its diagnostic specificity can be limited. Elastography, including both SE and SWE techniques, provides quantitative information on tissue stiffness, a valuable surrogate for tumor hardness often associated with malignancy, thereby improving diagnostic confidence. CEUS visualizes microvascular perfusion and hemodynamics, offering deep insights into tumor angiogenesis, which is a hallmark of cancer. However, CEUS involves contrast agent injection, is more operator-dependent, and has higher operational costs. The integration of multimodal ultrasound (e.g., BMUS + SWE + CEUS) in radiomics models leverages the strengths of each modality, often resulting in superior diagnostic performance compared to single-modality approaches. The choice of modality should therefore be guided by clinical context, available resources, and the specific diagnostic question at hand.

In summary, radiomics combined with ultrasound images could serve for elevating the diagnostic accuracy regarding breast lesions, and also can be used for reducing unnecessary biopsies.

## Predicting molecular subtypes of breast cancer

Breast cancer has 4 major subtypes: luminal A, luminal B, human epidermal growth factor receptor 2 enriched (HER2+), and triple-negative (26). Each subtype corresponds to unique clinical characteristics, treatment responses and survival outcomes (27). An accurate assessment of molecular subtypes well contributes to the personalized treatment and tumor prognosis. Immunohistochemistry (IHC) is a widely-used method for confirming molecular subtypes through biopsy. Nevertheless, as tumors exhibit spatial and temporal heterogeneity, a single biopsy with limited biopsy tissues may not represent the entire tumors (28). The accuracy in molecular subtyping depends on the biological distinctness of the subtypes and the richness of the input features. TNBC, often possessing distinct morphological features, is generally predicted with higher accuracy. The use of dynamic imaging, such as CEUS videos, provides supplementary information on tumor hemodynamics, which is particularly beneficial for distinguishing Luminal A and HER2+ subtypes that may appear similar on conventional ultrasound. Currently, researchers are attempting to use ultrasound radiomics as a noninvasive approach to make a precise prediction of breast cancer’s molecular subtypes (**Table 2**).

**Table 2:** Summary of ultrasound radiomics in predicting molecular subtypes of breast cancer.

Year	Authors	No. of lesions	Task	Modality	Result
2022	Wu et al. (29)	264	discriminating the luminal from non-luminal type	BMUS	AUC: 0.767
2023	Gong et al. (30)	170	predicting six categories: Luminal A, luminal B, HER2 overexpression, triple-negative, HR+, and HER2+	BMUS+CEUS	Accuracy: 70.2%, 69.7%, 84.0%, 86.9%, 74.5% and 72.5% respectively
2021	Jiang et al. (31)	2120	assessment of four breast cancer molecular subtypes: luminal A, luminal B, HER2+, triple-negative	BMUS	accuracy: form 80.07% to 97.02% for the test cohort A; and 87.94% to 98.83 for the test cohort B for each sub-category
2024	Xu et al. (34)	454	distinguish triple negative breast cancer from non-triple-negative breast cancer	BMUS	AUC: 0.813
2022	Du et al. (36)	360	differentiate triple-negative breast cancer from fibroadenoma	BMUS	AUC: 0.977
2023	Xu et al. (37)	359	predicting the expression of ER, PR, HER2 and Ki67	BMUS	AUC: 0.868, 0.811, 0.722 and 0.706 respectively
2022	Guo et al. (41)	309	predicting the expression of HER2	BMUS	AUC: 0.788
2023	Quan et al. (42)	445	predicting the expression of HER2	BMUS video	AUC: 0.810
2021	Cui et al. (44)	263	predicting the expression of Ki67 and P53	BMUS	AUC: 0.780 for Ki67; 0.710 for P53
2022	Wu et al. (45)	284	predicting the expression of Ki67	BMUS	AUC: 0.808
2023	Wang et al. (46)	231	predicting the expression of Ki67	BMUS	AUC: 0.880

BMUS, B-mode ultrasound; CEUS, contrast-enhanced ultrasound; HER2, human epidermal growth factor receptor 2; HR, hormone receptor; ER, estrogen receptor; PR, progesterone receptor; AUC, area under the curve.

In a prior study of Wu et al. that involved 264 patients, a radiomics score and a nomogram were built for discriminating luminal from non-luminal breast cancer (29). In the test cohort, the Rad-score and nomogram presented appreciable predictive results, and the AUC was 0.786 and 0.767 respectively. In addition, Gong and colleagues developed conventional ultrasound (CUS) and CEUS radiomics models to evaluate the relevance of radiomic features to cancer biologic features (30), who found that CEUS videos reflecting the dynamic blood perfusion information of tumors could provide auxiliary value to CUS radiomics in ascertaining luminal A and HER-2 overexpression (accuracy=70.2% and 84.0%). A deep convolutional neural network (DCNN) model by our team used ultrasound images from 1275 patients as the training samples to assess the tumors’ molecular subtypes (31). The above DCNN model achieved an accuracy from 80.07% (95% CI, 76.49–83.23%) to 97.02% (95% CI, 95.22–98.16%) and from 87.94% (95% CI, 85.0890.31%) to 98.83% (95% CI, 97.60–99.43) specific to the two test sets of four subtypes. The above studies showed that radiomics models derived from ultrasound images enable the identification of molecular subtypes with accuracy.

Triple-negative breast cancer (TNBC) is more biologically-aggressive, the recurrence rate is high and the survival outcomes are poor (32). A previous study shows that high-throughput quantitative ultrasonic characteristics could be used to predict the biological properties of TNBC (33). Xu and colleagues established a radiomics nomogram through integrating CUS features with Rad-score derived from grayscale ultrasound images to distinguish TNBC from non-TNBC, exhibiting a higher AUC value compared to the Rad-score model in the test dataset (0.813 vs 0.726) (34). Early studies suggested that specific to TNBC tumors, their shapes are regular and margins are circumscribed, also features usually encountered in fibroadenoma (35). Du et al. reported a study using ultrasound radiomic features together with clinical variables to differentiate TNBC from fibroadenoma, achieving a satisfactory discrimination ability (36). Ultrasound radiomic features can serve for improving the predictive accuracy of TNBC, which have the potential to improve patient life expectancy.

Moreover, Xu et al. built a radiomics model through a support vector machine, which could serve for confirming the molecular biomarker expression of breast cancer (37). The model displayed AUCs of 0.868, 0.811, 0.722 and 0.706 for ER, PR, HER2 and Ki67 in the validation cohort respectively. Patients who developed HER2-positive breast cancer tend to present a high rate of recurrence and poor prognosis (38), but can benefit from specific HER2-targeted therapies (39). In addition, HER2 expression was altered in 20-40% of patients undergoing NAC (40). Consequently, there is a need to establish an accurate, real-time and reproducible way to identify HER2 expression status. In a prior study of Guo et al., the radiomics signature was combined with a tumor size developed nomogram model for predicting the HER2 status of carcinoma (41), obtaining a slightly better predictive ability than the Rad-score model (AUC, 0.788 vs 0.786, in the validation set). Next, Quan and colleagues obtained good predictive performance in HER2 expression status through developing a deep learning radiomics (DLR) model based on ultrasound videos regarding breast lesions (42).

Ki-67 essentially markers tumor aggressive and proliferative activity (43), and p53 can suppress tumor development. Cui et al. reported that the quantitative radiomic features from ultrasound images were associated with of Ki-67 and p53 expressions (44). A prior study of Wu et al. that involved 284 patients developed a radiomics nomogram model through integrating ultrasound images with clinical risk factors for Ki-67 status determination, and the AUC reached 0.808 in the test set (45). In addition, Wang et al. developed a machine learning model utilizing the ultrasound radiomic features of intratumoral and peritumoral regions, and validated its high performance in identifying the Ki-67 expression (46).

In summary, the ultrasound radiomics model could reliably predict the molecule subtypes non-invasively, which is expected to help clinical decision-making to achieve a individual-based treatment for breast cancer patients.

## Predicting lymph node metastases of breast cancer

The axillary lymph node (ALN) status is adopted to effectively predict breast cancer prognosis, which can determine tumor stage and appropriate treatment strategies (47). Two primary measures for ALN status are sentinel lymph node biopsy (SLNB) and axillary lymph node dissection (ALND). Obviously, the above two methods may cause several postoperative complications (48). Hence, a non-invasive, accurate and efficient method shall be urgently proposed to predict ALN status. Ultrasound preoperatively assesses ALN status, but its diagnostic capability is limited (49). The predictive power for LN status is enhanced not only by the imaging modality but also by the model’s ability to capture tumor-host interactions. The consistent finding that peritumoral features improve prediction aligns with the biological understanding that lymphatic invasion and immune response occur at the tumor periphery. Deep learning models applied to elastography images appear to excel in this task, potentially because they can decode the complex relationship between primary tumor stiffness (a known predictor of metastasis) and LN involvement in a data-driven manner. Currently, more and more studies have suggested to apply the radiomics based on ultrasound images of primary breast tumors to confirm the ALN and SLN status (**Table 3**).

**Table 3:** Summary of ultrasound radiomics in predicting lymph node status of breast cancer.

Year	Authors	No. Of patients	Task	Modality	Result
2021	Zha et al. (50)	452	predicting SLN metastasis	BMUS	AUC: 0.833
2022	Bove et al. (51)	142	predicting ALN metastasis	BMUS	AUC: 0.886
2021	Lee et al. (52)	496	predicting ALN metastasis	BMUS	AUC: 0.831
2024	Zhang et al. (53)	755	predicting ALN burden	BMUS	AUC: 0.870 for N0 and N+(≥ 1) AUC: 0.911 for N+ (1-2) and N+(≥ 3)
2021	Lee et al. (54)	153	predicting ALN metastasis	BMUS	AUC: 0.805
2021	Zheng et al. (56)	584	predicting ALN burden	BMUS+SWE	AUC: 0.902 for N0 and N+(≥ 1) AUC: 0.905 for N+ (1-2) and N+(≥ 3)
2023	Wang et al. (57)	359	predicting SLN metastasis	BMUS+CDFI+elastography	DL-elastography model AUC: 0.876
2023	Wei et al. (62)	892	predicting ALN burden	BMUS	AUC: 0.920 for N0 and N+(≥ 1) AUC: 0.819 for N+ (1-2) and N+(≥ 3)
2024	Yao et al. (63)	278	predicting ALN burden	BMUS	AUC: 0.934 for N+(1-2) and N+(≥ 3)
2022	Jiang et al. (64)	433	predicting ALN burden	BMUS+SWE	C-index: 0.817 for N0 and N+(≥ 1) C-index: 0.810 for N+(1-2) and N+(≥ 3)

BMUS, B-mode ultrasound; SWE, shear wave elastography; CDFI, color doppler flow imaging; ALN, axillary lymph node; SLN, sentinel lymph node; AUC, area under the curve.

In most of the earliest studies, radiomic features obtained from gray-scale ultrasound images are used to predict LN status, with good performance (AUC values ranging from 0.833 to 0.886) (50–52). Lee et al. designed a radiomics model encompassing 23 features for confirming the ALN status, achieving better performance (AUC, 0.831 in the validation cohort) (52). Meanwhile, they also constructed a clinicopathologic model comprising 4 factors: tumor size, location, subtype and multiplicity, with an AUC of 0.708. Relative to the clinicopathological model, the radiomics model could more effectively predict ALN metastasis. Zhang et al. investigated 755 patients and assessed the predictive ability of internal and peritumoral ultrasound-based radiomics in predicting ALN status (53). They reported that intratumoral regions with a 3mm peritumoral region model exhibited the maximum assessment value, achieving an AUC of 0.903. Therefore, the surrounding tissue features of tumors are associated with ALN status, in tandem with previous findings of Lee et al. (54).

Ultrasound elastography is a new ultrasound technique to measure tissue stiffness, able to better identify malignant and benign breast lesions. According to studies,breast cancer lesion stiffness is a predictive factor of LNM (55). Thus, researchers innovatively explore whether radiomics models based on the elastography images can provide incremental value for predicting ALN metastasis. A study performed by Zheng et al. has shown that clinical parameters combined with radiomically-analyzed images of breast gray-scale ultrasound and SWE are capable of providing a noninvasive biomarker for the preoperative prediction of ALN metastasis (56). The method yielded a favorable diagnostic ability, and the AUC was 0.902 in the test cohort. Wang et al. adopted grayscale ultrasound, CDFI and elastography images to train DL radiomics models respectively, among which the DL-elastography model exhibited the best performance (57). Elastography images-based radiomics enhanced the predictive accuracy for ALN metastasis.

Of note, according to a trial, patients suffered clinical T1/T2 breast cancer with 1 or 2 sentinel LNs, showing an overall survival rate with SLN dissection (SLND) of not inferior than with ALN dissection (ALND) (58). Previous studies revealed that 40-65% of patients undergoing ALND as the results of SLNB were positive, without additional lymph nodes involvement (59, 60). The phenomenon, to some extent, is a significant overtreatment. Researchers further explored the radiomics approach to predict the ALNM burden. The confirmed histopathological diagnosis was taken into account to define the ALN status as low-burden (1–2 metastatic lymph nodes) or high-burden (≥ 3 metastatic) (61). Wei et al. (62) and Yao et al. (63) applied ultrasound radiomics combined with clinical factors for the preoperative prediction of ALNM burden in T1–2 breast cancer. Two models could be used for defining low and heavy metastatic ALN burdens, with an AUC reaching 0.819 and 0.934. The difference was that the former used a deep learning method and the latter used a machine learning classifier. Besides, our team built a nomogram incorporating radiomics signature taken from SWE images for predicting the number of ALN metastases, as well as verified its effect (64). The radiomics nomogram could be used to define low- and heavy-load ALNM while achieving a C-index of 0.810 in the validation cohort.

Taken together, the radiomics models integrating breast ultrasound images can effectively help clinicians to confirm the optimal axillary treatment.

## Predicting the efficacy of neoadjuvant chemotherapy for breast cancer

NAC usually serves for locally-advanced breast cancer (LABC), able to narrow breast lesion size, minimize the extent of surgeries and elevate the possibility for breast cancer patients to undergo breast-conserving surgeries (65). However, as lesions are heterogenous and complex, there is a considerable difference in patients’ response to NAC (66). Therefore, it is critical to accurately predict NAC tumor responses, which could provide benefits for poor NAC responders by timely adjusting the treatment regime. Ultrasound has become a routine imaging modality to be used repeatedly in the process of NAC (67). When a tumor has responses to chemotherapy, it is difficult to identify the changes within it or in its microenvironment in ultrasound images. Several studies have shown that radiomics can capture subtle changes in ultrasound images, with good performance for predicting pathologic responses to NAC (**Table 4**).

**Table 4:** Summary of ultrasound radiomics in predicting the response of NAC.

Year	Authors	No. of patients	Task	Modality	Result
2020	DiCenzo et al. (68)	82	predicting the response to NAC	BMUS	accuracy: 87%
2023	Yu et al. (69)	603	predicting the response to NAC	BMUS	AUC: 0.939
2021	Osapoetra et al. (70)	78	predicting the response to NAC	BMUS	AUC: 0.860
2023	Huang et al. (71)	255	predicting the response to NAC	BMUS+SWE	AUC: 0.780
2022	Gu et al. (72)	168	predicting the response to NAC	BMUS	AUC: 0.812 (after second course of NAC) AUC: 0.937 (after fourth course of NAC)
2024	Huang et al. (73)	112	predicting the response to NAC	BMUS+SWE	AUC: 0.91 (after two cycle of NAC) AUC: 0.94 (after four cycle of NAC)
2023	Zhang et al. (74)	525	predict the response of patients with ALN positive breast cancer to NAC	BMUS	AUC: 0.858
2023	Gu et al. (75)	484	predicting the status of tumors and lymph node metastasis after NAC	BMUS	AUC: 0.896 for predicting the status of tumors AUC: 0.863 for predicting the status of lymph node

BMUS, B-mode ultrasound; SWE, shear wave elastography; NAC, neoadjuvant chemotherapy; AUC, area under the curve.

In studies of DiCenzo et al. (68), quantitative ultrasound radiomic features could serve for forecasting responses to NAC with excellent performance. In a multicenter study by Yu et al., a DL radiomics (DLR) model that integrated pretreatment ultrasound images and clinical-pathologic factors was proposed for forecasting the NAC responses in breast cancer (69). The DLR model achieved a satisfactory prediction, and the AUC reached 0.939 in the validation cohort, outperforming two radiologists’ predictions (p < 0.05). Furthermore, Osapoetra et al. built a model with quantitative ultrasound and texture-derivate features extracted from tumor cores and 5-mm tumor margins (70). The model based on the SVMRBF classification algorithm can be used to make an optimal separation between responders and non-responders to NAC. Huang et al. analyzed 255 patients receiving NAC, meanwhile both radiomics and CNN models that integrated SWE images could more effectively predict NAC responses compared to models based on grayscale ultrasound images (71). In addition, the CNN model combining multimodal ultrasound images was the best predictor. Based on the above evidence, radiomics based on ultrasound images, including B-mode ultrasound and SWE, may be a valuable biomarker to evaluate NAC responses.

To further improve breast cancer patients’ early prediction performance of NAC response, researchers attempted to construct radiomics models integrating ultrasound images at different courses of NAC treatment (72, 73). Gu et al. built two DL radiomics models, DLR-2 and DLR-4, based on ultrasound images obtained before NAC, after the second course and after the fourth course, achieving an AUC of 0.812 and 0.937 (72). Meanwhile, they further proposed a DLR pipeline combined with DLR-2 and DLR-4 to stepwise predict the early response to NAC, by which 90% of non-responders were identified and they might benefit greatly from the adjustment of treatment strategies. A study developed a model incorporating the radiomic features of serial BMUS and SWE images throughout the NAC course, achieving excellent performance in predicting early pathological responses (73). Thus, the radiomic features from ultrasound images during the course of NAC are beneficial for predicting NAC responses.

The ALN status following NAC is a valuable reference for axillary treatment decision making. To comprehensively assess the NAC performance, researchers have paid attention to the ALN status evaluation after NAC using noninvasive radiomics methods. Zhang et al. (74) applied ALN ultrasound images and Gu et al. (75) used pre-NAC and after-NAC breast ultrasound images to develop radiomic nomograms with clinical factors to predict ALN status. The results confirmed that most non-LNM patients after NAC were recognized precisely, with unnecessary axillary surgeries avoided.

According to previous studies, radiomics models combining ultrasound images could help to confirm the breast cancer patients’ response to NAC as a non-invasive objective biomarker. Prospective research accompanied by massive data is necessary for validating models’ clinical usefulness. Currently, clinicians still need to make a comprehensive assessment combining different resources and patient demands, thereby confirming whether the patient should be discontinued from NAC.

## Predicting the prognosis of breast cancer

Ultrasound morphological features are related to prognostic factors in breast cancer (76). Based on this foundation, researchers are actively exploring whether quantitative radiomic features from ultrasound images are the potential biomarkers for predicting prognosis (**Table 5**).

**Table 5:** Summary of ultrasound radiomics in predicting the prognosis of breast cancer.

Year	Authors	No. of patients	Task	Modality	Result
2021	Xiong et al. (77)	620	Predict disease-free survival (DFS) of invasive breast cancer	BMUS	C-index: 0.796
2021	Dasgupta et al. (78)	83	Predict recurrence for patients with locally advanced breast cancer	BMUS	AUC: 0.76
2021	Yu et al. (79)	486	Predicting DFS after resection of triple negative breast cancer	BMUS	C-index: 0.75

BMUS, B-mode ultrasound; DFS, disease-free survival; AUC, area under the curve.

Xiong et al. identified the clinical value of nomograms containing the ultrasound radiomics signature in forecasting the disease-free survival (DFS) regarding invasive breast cancer, and the C-index reached 0.796 in the validation cohort (77). Dasgupta et al. integrated pre-treatment ultrasound images into a radiomics model for ascertaining the recurrence among patients suffering locally-advanced breast cancer, and the model accuracy reached 82% (78). The above studies have suggested favorable predictive analyses on the prognosis of breast cancer using intratumoral radiomic features extracted from ultrasound images, but they have neglected the fact that the peritumoral regions may contain important information related to prognosis. In a multi-institutional study including 486 patients, Yu and colleagues developed a nomogram with features extracted from breast tumors and peritumoral regions for predicting DFS after the resection of TNBC, and its external validation C-index reached 0.75 (79). The nomogram exhibits a better predictive ability than clinicopathological models.

In summary, ultrasound radiomic features are a potential imaging predictor for breast cancer patients’ prognosis, while radiomics models hold promise to improve individualized prognosis evaluation and assist clinicians in developing personalized treatment.

## Challenges and perspectives

Radiomics is an emerging filed that aims to transform high-throughput features extracted from medical images into quantitative information. Ultrasound radiomics has obtained many promising results in diagnosing breast cancer and predicting prognosis. Nevertheless, challenges still exist when breaking the “laboratory” effect and applying radiomics models in clinical practice.

First of all, the vast majority of studies were small-sample single-center retrospective analyses, retrospective analyses lead to a partial loss of sample size and patient information, while small samples can lead to an over-extraction of features, which may result in deficient stability of ultrasound radiomics models. Therefore, future prospective experiments with large multi-center samples shall be conducted for validating as well as enhancing the model robustness. Besides, the differences in ultrasound instruments and the lack of uniform standards for operating procedures, as well as whether the predicted results of models are influenced by these factors, need to be further verified, making it difficult to acquire standardized multi-center data.

To address the critical issue of data standardization, future multi-center collaborations must prioritize the development and adoption of unified image acquisition protocols. These protocols should specify standardized settings for transducer frequency, gain, dynamic range, and scanning procedures to ensure the homogeneity and reproducibility of radiomics features across different institutions and device vendors. Concurrently, the ‘black box’ nature of complex deep learning models remains a significant barrier to clinical trust and adoption. Integrating eXplainable AI (XAI) techniques, such as SHAP (SHapley Additive exPlanations) and LIME (Local Interpretable Model-agnostic Explanations), can help elucidate the contribution of specific image features to the model’s prediction, thereby enhancing transparency and fostering clinician confidence.

In addition, the image segmentation of traditional radiomic studies is manual and the differences will be among operators, which increases the uncertainty of the results. DL can be used to integrate feature extraction process that can autonomously learn and extract disease-relevant features through complex multi-layer neural networks, without human involvement. However, another challenge is that DL method lacks transparency and interpretability, also the so-called ‘black box’, meaning that the relevance of extracted image features through DL method to tumor biological information is not well understood. This greatly affects physicians’ trust in DL radiomics models. In summary, it needs to build more interpretable DL radiomics models.

Currently, multi-omics research has become a hot topic in breast cancer (80). In a multi-center radio-multiomics study, Su et al. revealed intratumor heterogeneity phenotypes and therapeutic targets using comprehensive analysis radiomics, genomic, transcriptomic, metabolomic and pathological data (80). Thus, multi-omics research may accelerate breast cancer personalize diagnosis, decision making and prediction, which may become a major direction for future research.

Looking forward, the clinical integration pathway for ultrasound radiomics models is more likely to be as a decision-support tool rather than a fully autonomous decision-maker. These models can assist radiologists and oncologists by providing a quantitative ‘second opinion,’ particularly in challenging cases such as classifying BI-RADS 4 lesions or predicting NAC response early. Future research should focus on embedding these models into hospital Picture Archiving and Communication Systems (PACS) and Clinical Decision Support Systems (CDSS) for seamless workflow integration. Furthermore, exploring ‘delta-radiomics’ (changes in features over time) and multi-omics integration—correlating radiomic features with genomic, transcriptomic, and pathologic data—holds immense promise for uncovering novel biomarkers and achieving truly personalized medicine in breast cancer care. Finally, there is a pressing need to extend ultrasound radiomics research to special populations, such as patients with gestational breast cancer, where avoiding radiation is paramount, and elderly patients with comorbidities, who may have distinct tumor biology and treatment responses.

## Conclusion

Ultrasound radiomics has yielded many impressive results in diagnosing benign and malignant breast lesions, predicting molecular subtypes, evaluating LN status, predicting NAC responses and disease prognosis. Furthermore, radiomics models based on multimodal ultrasound images and those combining radiomics features with clinical-pathologic factors can be used to perform the above tasks more accurately. A future research direction lies in developing multi-omics models which integrate clinical data, microscopic genetic data and ultrasound images of multi-center patients. Currently, the non-invasive ultrasound radiomics model is promisingly instrumental in the individual-based treatment which might be used in clinics in the near future to benefit patients with breast cancer.
